# Genetic polymorphism in *Leishmania infantum* isolates from human and animals determined by *nagt* PCR-RFLP

**DOI:** 10.1186/s40249-018-0439-y

**Published:** 2018-06-14

**Authors:** Adil El Hamouchi, Sofia El Kacem, Rajaa Ejghal, Meryem Lemrani

**Affiliations:** Laboratory of Parasitology and Vector-Borne-Diseases, Institut Pasteur du Maroc, Casablanca, Morocco

**Keywords:** *Leishmania infantum*, Genetic variability, N-acetylglucosamine-1-phosphate transferase, PCR-RFLP, Morocco

## Abstract

**Background:**

*Leishmania infantum* is the causative agent of human visceral leishmaniasis (VL) and sporadic human cutaneous leishmaniasis (CL) in the Mediterranean region. The genetic variation of the *Leishmania* parasites may result in different phenotypes that can be associated with the geographical distribution and diversity of the clinical manifestations. The main objective of this study was to explore the genetic polymorphism in *L. infantum* isolates from human and animal hosts in different regions of Morocco.

**Methods:**

The intraspecific genetic variability of 40 Moroccan *L. infantum* MON-1 strains isolated from patients with VL (*n* = 31) and CL (*n* = 2) and from dogs (*n* = 7) was evaluated by PCR-RFLP of *nagt*, a single-copy gene encoding N-acetylglucosamine-1-phosphate transferase. For a more complete analysis of *L. infantum* polymorphism, we included the restriction patterns of *nagt* from 17 strains available in the literature and patterns determined by in-silico digestion of three sequences from the GenBank database.

**Results:**

Moroccan *L. infantum* strains presented a certain level of genetic diversity and six distinct *nagt*-RFLP genotypes were identified. Three of the six genotypes were exclusively identified in the Moroccan population of *L. infantum*: variant M1 (15%), variant M2 (7.5%), and variant M3 (2.5%). The most common genotype (65%), variant 2 (2.5%), and variant 4 (7.5%), were previously described in several countries with endemic leishmaniasis. Phylogenetic analysis segregated our *L. infantum* population into two distinct clusters, whereas variant M2 was clearly distinguished from both cluster I and cluster II. This distribution highlights the degree of genetic variability among the Moroccan *L. infantum* population.

**Conclusion:**

The *nagt* PCR-RFLP method presented here showed an important genetic heterogeneity among Moroccan *L. infantum* strains isolated from human and canine reservoirs with 6 genotypes identified. Three of the six Moroccan *nagt* genotypes, have not been previously described and support the particular genetic diversity of the Moroccan *L. infantum* population reported in other studies.

**Electronic supplementary material:**

The online version of this article (10.1186/s40249-018-0439-y) contains supplementary material, which is available to authorized users.

## Multilingual abstracts

Please see Additional file 1 for translation of the abstract into the five official working languages of the United Nations.

## Background

*Leishmania infantum*, a flagellated protozoan in the Trypanosomatidae family, is the causative agent of human visceral leishmaniasis (VL) and sporadic human cutaneous leishmaniasis (CL) in the Mediterranean region [1–3]. In Morocco, and all around the Mediterranean basin, *L. infantum* MON-1 is the predominant causative agent of VL, with the domestic dog as the main reservoir and *Phlebotomus perniciosus, Phlebotomus ariasi,* and *Phlebotomus longicuspis* as the vectors [4–7]. The zymodeme MON-24, mainly considered to cause CL, has been detected in both humans and dogs in Morocco [8]. VL is endemic in the northern part of the country, but sporadic cases have been reported in the south [9]. An average of 128 VL cases were reported every year from 2008 to 2013 with a predominance of cases in children aged under 10 years [10]. The annual incidence rate of VL is estimated to be 0.4 cases per 100 000 people [11]. In recent years, changes in the epidemiology of *L. infantum* have been reported in Morocco, including a southward spread of the parasite, a new focus of CL, and the appearance of treatment-resistant *L. infantum* [12, 13]. Several studies investigating the potential contribution of the parasite to the clinical pleomorphism of leishmaniasis have shown a correlation between specific *Leishmania* genotypes and clinical forms, and demonstrate that parasite genotyping plays an essential role in both clinical and epidemiological risk assessment [14]. A sound and consensual taxonomical background based on the knowledge of the phylogenetic diversity of *Leishmania* is needed for a better understanding of epidemiological changes [15]. The broad epidemiological and clinical diversity of *L. infantum* raises the need of analysis of genetic variability. To date, several methods using powerful molecular tools have been employed for typing *Leishmania* strains [16, 17]. Multilocus enzyme electrophoresis (MLEE) is the most commonly used technique for *Leishmania* typing. However, this technique requires cultivation of the parasite and cannot always discriminate between closely related strains. Different PCR-based methods have been employed to ascertain the intraspecific genetic variability of *Leishmania* and have contributed to the development of epidemiological studies [16, 17]. PCR amplification of kinetoplast DNA (kDNA) minicircles followed by analysis of restriction fragment length polymorphism (RFLP) has shown the genetic diversity between closely related strains of *L. infantum* MON-1 [18–20]. Further genetic diversity within *Leishmania* strains has been elucidated by random amplification of polymorphic DNA (RAPD) [21, 22], PCR-RFLP of the antigen-encoding genes *gp*63 and *cpb* [23, 24], sequence analysis of intergenic spacer regions (ITS) [25, 26], multilocus microsatellite typing (MLMT) [18, 27–29], and multilocus sequence typing (MLST) [30–32]. PCR-RFLP of the single-copy gene, *nagt*, which encodes N-acetylglucosamine-1-phosphate transferase (NAGT), has been used for intraspecies divergence analyses of *Leishmania* spp. [33, 34]. Analysis of the *nagt* sequence revealed the existence of five different genotypes within a population of 86 *L. infantum* isolates from distinct regions [34]. The *nagt* gene is highly conserved and functionally indispensable. Therefore, we have used PCR-RFLP of *nagt* gene as a molecular method to analyze genetic variability within a population of Moroccan *L. infantum* isolated from humans and canine reservoirs.

## Methods

### Leishmania infantum strains

**Fig. 1:**
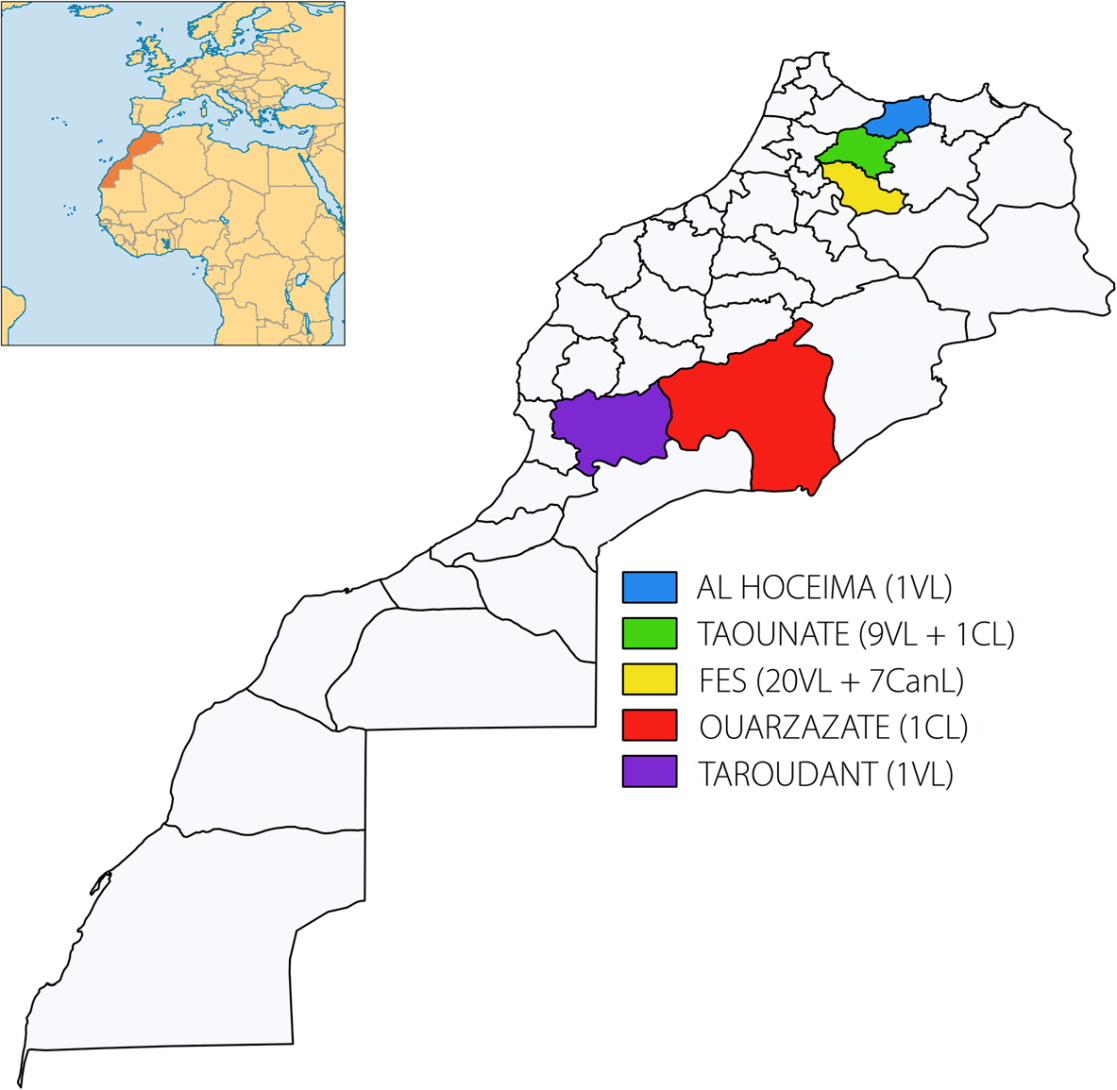
Number and geographical distribution of Moroccan *L. infantum* isolates in this study (Number between brackets indicates the number of samples; VL: Visceral leishmaniasis; CL: Cutaneous leishmaniasis; CanL: Canine leishmaniasis)

A total of 40 clinical isolates of *L. infantum* were used. The strains were isolated in Novy-MacNeal-Nicolle culture medium. Thirty-three were isolated from immunocompetent patients with VL (*n* = 31) and CL (*n* = 2); seven strains were isolated from canine reservoirs. The samples were collected from the northern VL endemic foci of Morocco in the provinces of Fes (*n* = 27), Taounate (*n* = 10) and Al Hoceima (*n* = 1), as well as from some VL sporadic areas in the southern provinces of Ouarzazate (*n* = 1) and Taroudant (*n* = 1). The clinical form, geographical distribution and the number of the studied isolates are presented in Fig. 1. All VL and canine leishmaniasis (CanL) strains were previously characterized by MLEE as belonging to zymodeme MON-1, except one CL strain collected from Taounate, which belongs to zymodeme MON-24. The CL strain from Ourzazate province was not characterized.

### DNA extraction

DNA was extracted from promastigote pellets using the PureLink® Genomic DNA Mini Kit (Invitrogen, Carlsbad, CA, USA), according to the manufacturer’s instructions. A final volume of 100 μl of DNA was obtained and stored at − 20 °C until use.

### PCR amplification and RFLP analysis

The ~ 1.4-kb *nagt* gene amplifications were performed with the primer pair L1 (5’-TCATGACTCTTGGCCTGGTAG-3′) and L4 (5’-CTCTAGCGCACTTCATCGTAG-3′), as previously described by Akman et al. [33], with some modifications. PCR mixtures consisted of between 50 and 100 ng of DNA, 1.25 U of GoTaq® DNA polymerase (Promega, Madison, WI, USA), 1X PCR buffer, 0.4 μmol/L of each primer, 1.5 mmol/L MgCl_2_ and 0.2 mmol/L of each dNTP. The thermocycler settings were an initial denaturation at 95 °C for 5 min, 30 cycles at 94 °C for 60 s, 58 °C for 60 s, and 72 °C for 90 s, and a final extension step at 72 °C for 5 min. Further RFLP analysis of the PCR-amplified *nagt* gene was performed separately using three restriction enzymes: *Nae*I, *Alw*I, and *Nci*I (New England Biolabs, Ipswich, MA, USA). After conditions optimization, digestion reactions were carried out in a final volume of 10 μl including 5 μl of PCR product, 10 U of restriction enzyme, and 1× recommended buffer for each enzyme. All restriction reactions were incubated overnight at 37 °C. The restriction fragments were resolved by electrophoresis for 2–3 h on a 3% agarose gel containing ethidium bromide (0.5 μg/ml) and visualized under UV illumination.

### Selection of *nagt* sequences from GenBank database and in-silico RFLP analysis

The Primer-BLAST tool from NCBI was used to search *nagt* sequences corresponding to the *L. infantum* species. Briefly, the sequences of the primer pair L1/L4 were blasted, and only *nagt* sequences with nearly 100% homology with the primers and belonging to *L. infantum* species were chosen. Three *nagt* sequences, approximately 1405 bp, were selected and analyzed by in-silico digestion with the same restriction enzymes using the option “Find restriction sites” in Unipro UGENE 1.25 [35]. Characteristics of the three selected sequences are shown in Table 1.

**Table 1:** Sequences of *nagt* from the GenBank database

Species	WHO code ^a^/strain ^b^	Country	Accession number (Selected region)
*L. infantum*	MCAN/ES/98/LIM-877 ^a^	Spain	KU680842.1
*L. infantum*	MHOM/IR/04/IPI-UN ^a^	Iran	KU680843.1
*L. infantum*	JPCM5 ^b^	Spain	FR796468.1 (1589080–1 590 484)

^a^WHO code

^b^Strain name

### Phylogenetic analysis

The restriction patterns resulting from the digests of the *nagt* PCR products by the 3 tested endonucleases and those obtained through in-silico digestion were used to identify genotypes of the Moroccan *L. infantum* isolates and the strains selected from GenBank databse, respectively. The genotypes identified in this work were named according to Waki et al. [34]. To analyze the phylogenetic relationships, the 40 Moroccan isolates of this study (Fig. 1) and the 3 samples selected from Genbank database (Table 1) as well as 17 strains previously described by Waki et al. [34] (Additional file 2: Table S1), forming a database of 60 samples. Subsequently, the *nagt* restriction patterns from all samples were inserted into a binary matrix, with the restriction sites coded as present (1) or absent (0). Phylogenetic analysis was performed using the package PHYLIP 3.69 [36]. The binary matrix was converted into a distance matrix with the Restdist tool and the resulting distance matrix was used to construct a rooted tree based on the Unweighted Pair Group Method with Arithmetic mean (UPGMA) algorithm with the Neighbor tool. The robustness of the phylogenetic analysis and significance of the branch order were determined by bootstrap analysis carried out on 100 replicates using SEQBOOT program, provided with the PHYLIP package.

## Results

### PCR-RFLP analysis of *nagt*

**Fig. 2:**
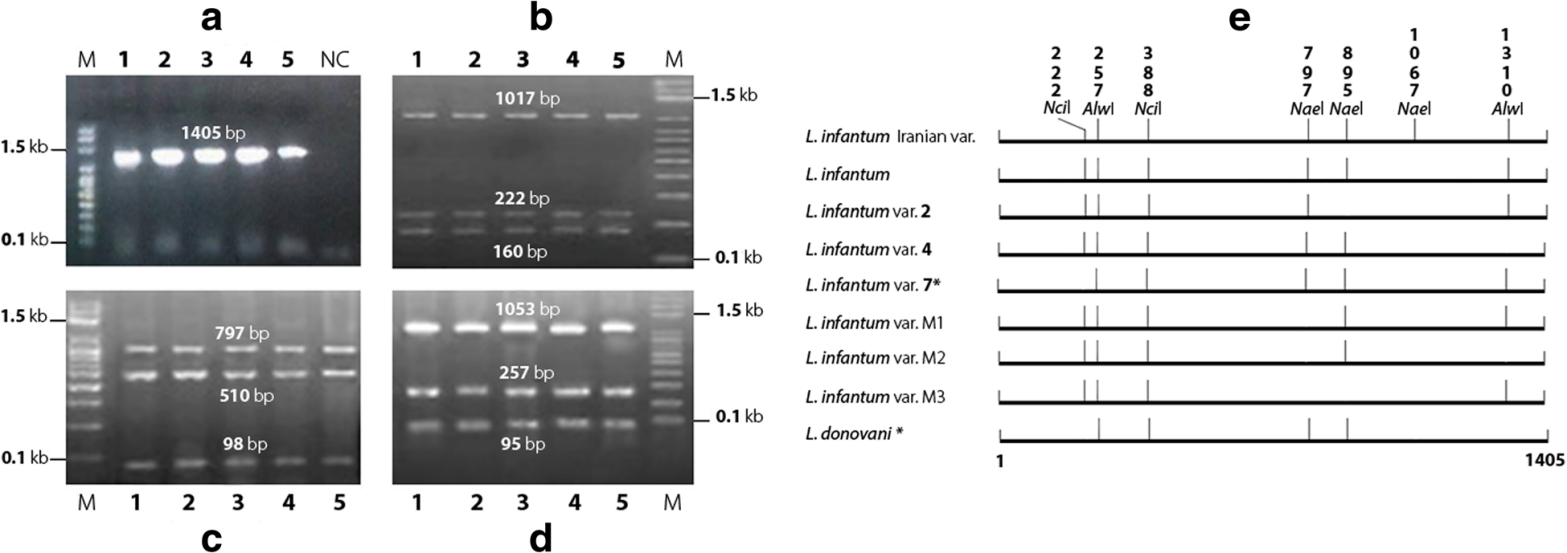
**a** PCR amplification of *nagt* gene (1.4 kb) from Moroccan *L. infantum* strains. **b**, **c**, **d** PCR-amplified *nagt* digested with *Nci*I, *Nae*I, and *Alw*I, respectively. The numbers above and below the digested fragments indicate their sizes. Lanes 1–5: DNA samples of Moroccan *L. infantum* isolates 1 to 5; NC: Negative control; M: Molecular marker HyperLader™ 100 bp plus (Bioline, Taunton, MA, USA). **e** RFLP maps illustrating the *nagt* genotype identified in this work and those identified by Waki et al. [34]*. The vertical numbers indicate the cut position of each enzyme

PCR amplification of *nagt* from 40 Moroccan *L. infantum* strains produced a DNA fragment with the expected size of about 1.4 kb (Fig. 2a). *nagt*-RFLP analysis of PCR products with three endonucleases revealed genetic heterogeneity among the Moroccan *L. infantum* population. Digestion with *Alw*I and *Nae*I produced two and four different patterns, respectively. The endonuclease *Nci*I resulted in a monomorphic pattern (Table 2). To discriminate genotypes, the RFLP patterns of each strain were compared with *L. infantum nagt* restriction maps [34]. The *L. infantum* strain JPCM5 (Accession number: FR796468.1) was used as a reference sequence to identify the *nagt* restriction sites of the three tested enzymes and to confirm the size of restriction fragments. The cut positions on the reference sequence corresponding to each enzyme are shown in additional Additional file 3: Table S2. Representative RFLP patterns for *Alw*I, *Nci*I, and *Nae*I are shown in Fig. 2b, c, and d.

**Table 2:** Fragment sizes obtained from restriction digest

Enzymes	*AlwI*		*NaeI*					*NciI*
Patterns	a	b	a	b	c	d	e^a^	a
Fragments (bp)	1053 257 95	1148 257	797 510 98	797 608	895 510	1405	797 338 172 98	1017 222 166

^a^RFLP in-silico analysis of *nagt* of *L. infantum* strain MHOM/IR/04/IPI-UN10 (KU680843.1)

### Identification of genotypes

Analysis of *nagt*-RFLP restriction patterns allowed grouping of the 40 Moroccan isolates of *L. infantum* into six distinct genotypes (Table 3) when compared to *nagt* genotypes previously established for the *L. donovani* and *L. infantum* species [34]. Of the six genotypes identified, common *L. infantum* and *L. infantum* variants 2 and 4 had been previously described [34]. The other three genotypes were exclusively identified in this study of Moroccan *L. infantum* and named M1, M2, and M3. The predominant *L. infantum* in this study was the common genotype with a proportion of 65% (26/40), followed by *L. infantum* variant M1 at 15% (6/40), variant 4 and variant M2 at 7.5% each (3/40 each), and one each of variant 2 and variant M3 (2.5% each). The 32 *L. infantum* isolates causing VL were grouped into five genotypes, whereas strains causing CanL (*n* = 7) belonged to the common genotype. The dermotropic strain isolated from the south of Morocco had a specific *nagt* genotype and was named variant M3. However, the dermotropic strain originating from the north of Morocco had the common *nagt* genotype. An additional *nagt* genotype was identified among three *nagt* sequences retrieved from the GenBank database by *in-silico* RFLP analysis (Table 2). Of the three sequences, the variant causing VL is from Iran and was designated as the “Iranian variant” (Table 3). The other two *L. infantum* sequences correspond to the predominant *nagt* genotype. Restriction maps were constructed to clearly illustrate the differences between the *nagt* genotypes identified here and in the 2007 study by Waki et al. [34] (Fig. 2e).

**Table 3:** *nagt* genotypes identified within Moroccan *L. infantum* population and defined by the restriction patterns obtained with endonucleases *Alw*I, *Nae*I and *Nci*I

Genotype	RFLP patterns			Strains
	*AlwI*	*NaeI*	*NciI*	
*L. infantum* (common genotype)	a	a	a	26 (18 VL + 7 CanL +1CL)
*L. infantum* variant 2	a	b	a	1 (VL)
*L. infantum* variant 4	b	a	a	3 (VL)
*L. infantum* variant M1	a	c	a	6 (VL)
*L. infantum* variant M2	b	c	a	3 (VL)
*L. infantum* variant M3	a	d	a	1 (CL)
*L. infantum* Iranian variant^a^	a	e	a	1 (VL)

*VL* Visceral leishmaniasis, *CL* Cutaneous leishmaniasis, *CanL* Canine visceral leishmaniasis

^a^This variant was identified by in-silico RFLP analysis of an Iranian sample retrieved from the GenBank database

### Phylogenetic analysis

**Fig. 3:**
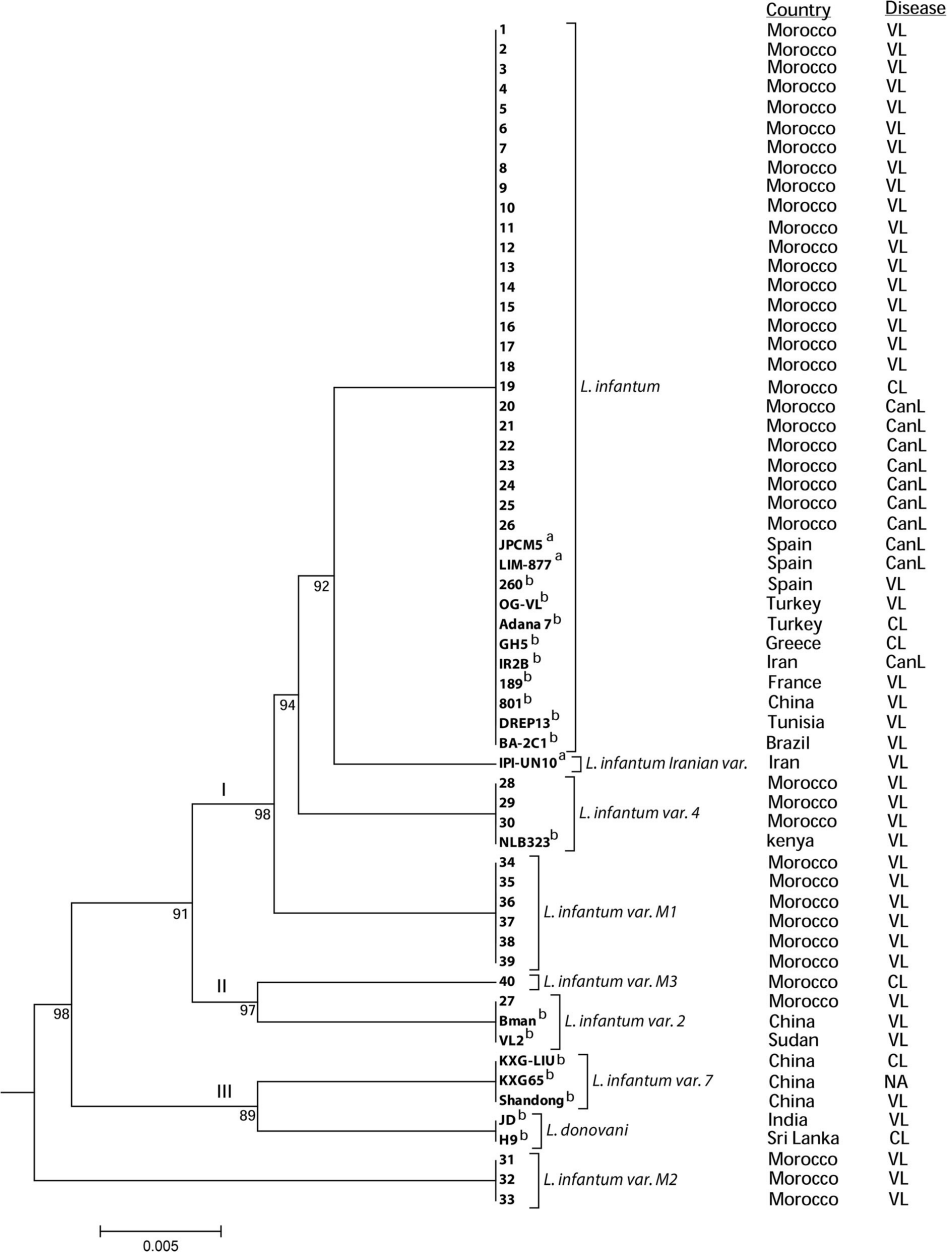
UPGMA tree constructed from nagt PCR-RFLP data of *L. infantum* (*n* = 58) and *L. donovani* (*n* = 2). The strains numbered from 1 to 40 correspond to Moroccan *L. infantum* isolates; ^a^Isolates retrieved from GenBank database; ^b^Isolates described by Waki et al. [34]. Bootstrap values (out of 100 replicates) are mentioned below the nodes

The UPGMA dendrogram, inferred from the genetic distances calculated from the *nagt* PCR-RFLP data, allowed an overall and clear visualization of the relationships between the nine *nagt* genotypes identified (Fig. 3). The UPGMA tree topology was supported by highly significant bootstrap values (> 90%). Phylogenetic analysis of the 60 samples showed that most of the isolates (37/60) correspond to the common *L. infantum* genotype, regardless of geographical origin, host, or clinical forms. Except for variant M2, the *L. infantum* variants were grouped into three clusters (Fig. 3). The largest, cluster I, included strains from Mediterranean countries (Morocco, Spain, France, Tunisia, Greece, and Turkey), East African countries (Sudan and Kenya), Asian countries (Iran and China), and Brazil. Cluster II was composed of strains isolated from Morocco, Sudan, and China. However, cluster III consisted of strains solely originating from Asia: two *L. donovani* strains each isolated from India and Sri Lanka and three Chinese *L. infantum* variant 7 strains, exclusively identified in Chinese isolates. There was no structuring of the strains according to their hosts (human or dog) or clinical forms (CL or VL).

## Discussion

*nagt* is a highly conserved, single-copy gene, which encodes the endoplasmic reticulum trans-membrane protein, N-acetylglucosamine-l-phosphate transferase. The stability of the *Leishmania* virulence factor, zinc-metalloprotease GP63, is associated with N-glycosylation, which is dependent on N-acetylglucosamine-1-phosphate transferase catalyzing the first step [37].

In this study, we investigated the genetic variability of 40 Moroccan *L. infantum* strains isolated from canine reservoirs and immunocompetent patients with VL and CL. PCR-RFLP analysis of *nagt* showed important genetic heterogeneity among Moroccan *L. infantum* strains. Six different *nagt* genotypes were identified and the common *nagt L. infantum* genotype was predominant in our samples (65%, 26/40). The common genotype was also reported as the most frequent genotype (73%, 63/86) in a worldwide population of *L. infantum* collected from Brazil, southern European countries, Tunisia, Iran, and China [34]. Taken together, these data indicate that the *L. infantum* common *nagt* variant is the most widespread genotype in different foci of VL worldwide. Moreover, this predominant genotype included samples isolated from human CL and VL patients and canine reservoirs, illustrating the zoonotic cycle of transmission of *L. infantum* [38, 39]. One of the isolated strains was characterized as *nagt* variant 2 and three strains were characterized as variant 4. Those genotypes were previously reported in small numbers in Sudan and China for variant 2 and in Kenya for variant 4 [34]. These findings suggest that variant 4 may be an African genotype as opposed to *L. infantum* variant 7, which seems to be exclusive to Chinese isolates [34].

The Moroccan *L. infantum* genotypes, variants M1, M2, and M3, have not been previously described. Variants M1 and M2 were isolated from VL patients and variant M3 was from a CL patient. Phylogenetic analysis segregated variants M1 and M3 into two distinct clusters (I and II, respectively). However, variant M2 was clearly distinguished from the three clusters obtained. This distribution highlights the important degree of genetic variability among the Moroccan *L. infantum* population. Among the three clusters, there was no association between geographical origin of isolates or the disease forms (VL/CL). The lack of association between *Leishmania nagt* gene-based grouping and VL/CL disease phenotype of isolates had been previously reported [34]. We also identified a new *nagt* genotype, the “Iranian variant.” This unique variant, belonging to cluster I, is the most homologous to the predominant genotype. Additionally, *L. donovani* species presented a unique *nagt* restriction profile, which corroborates that *L. donovani* and *L. infantum* are genetically different [40]. Chinese *L. infantum* variant 7 isolates are grouped in cluster III with *L. donovani* from India and Sri Lanka, two Asian neighboring countries. These results align with a previous report that a group of Chinese *L. infantum* strains were closely related to *L. donovani* strains from India [41]. Other studies have also reported that some *L. infantum* strains are more closely related to *L. donovani* than other *L. infantum* strains [42].

## Conclusions

This work showed important intraspecific genetic variability among Moroccan *L. infantum* strains. Three of the six *nagt* variants are exclusive to Moroccan isolates. The *nagt* PCR-RFLP method used here seems to have a good resolving power and supports the substantial genetic diversity of the Moroccan *L. infantum* population reported in other studies [20, 27], thus confirming its usefulness. However, further studies should be carried out by extending the *nagt* PCR-RFLP method to a larger number of strains representing different hosts (human, dogs and phlebotomine sand fly), geographical areas and zymodemes types, to better understand the molecular epidemiology of *L. infantum* in Morocco and the other endemic countries.

## Additional files

Additional files 1: Multilingual abstracts in the five official working languages of the United Nations. (PDF 1009 kb)

Additional files 2: **Table S1.** List of strains of *L. infantum* and *L. donovani* previously genotyped as *nagt* variant by Waki et al. [34] and used in this work. (DOCX 13 kb)

Additional files 3: **Table S2.** Cut positions of restriction enzymes tested by in-silico digestion of *nagt* (1405 bp) of *L. infantum* JPCM5. (DOCX 12 kb)

## Abbreviations

CanL: Canine leishmaniasis
CL: Cutaneous leishmaniasis
*nagt*: N-acetylglucosamine-1-phosphate transferase
VL: Visceral leishmaniasis
WHO: World Health Organization
