# Supplementary material for: Genetic polymorphism in Leishmania infantum isolates from human and animals determined by nagt PCR-RFLP
Source: Infect Dis Poverty. 2018 Jun 14;7:54. doi: 10.1186/s40249-018-0439-y (PMC6001066; doi:10.1186/s40249-018-0439-y)
Supplement: Supplementary file 1 — Multilingual abstracts in the five official working languages of the United Nations. (PDF 1009 kb) [file 40249_2018_439_MOESM1_ESM.pdf]

Translation of the abstract into the five official working languages of the United Nations

تعدد الأشكال الوراثية في عزلات الليشمانيا الطفلية من الإنسان والحيوانات التي تم تحديدها بواسطة تفاعل البوليميراز المتسلسل - تقييد طول الجزء متعدد الأشكال nagt RFLP-PCR

قدمه: عادل الحموشي، صوفيا القاسم، رجاء إجغال وميريم ليمراني

الملخص

المعلومات الأساسية: الليشمانيا الطفلية هي العامل المسبب لداء الليشمانيات الحشوي البشري (VL) وداء الليشمانيات الجلدي البشري المتفرق (CL) في منطقة البحر الأبيض المتوسط . قد يؤدي الاختلاف الجيني لطفيليات الليشمانيا إلى أنماط ظاهرية مختلفة يمكن أن ترتبط بالتوزيع الجغرافي وتنوع المظاهر السريرية. كان الهدف الرئيسي من هذه الدراسة هو استكشاف تعدد الأشكال الوراثي في عزلات الليشمانيا الطفلية من مضيفي الإنسان والحيوان في مناطق مختلفة من المغرب.

الأساليب: تم تقييم التباين الوراثي بين الأنواع لـ 40 سلالة *1-MON* ليشمانيا طفلية مغربية معزولة من المرضى الذين يعانون من الليشمانيات الحشوية البشرية VL (n = 31) والليشمانيات الجلدية البشرية المتفرقة CL (n = 2) ومن الكلاب (n = 7) من خلال تفاعل البوليميراز المتسلسل - تقييد طول الجزء متعدد الأشكال RFLP-PCR لـ nagt ، وهو جين نسخة أحادية لترميز osphateph-1-acetylglucosamine-N. للحصول على تحليل أكثر اكتمالاً لتعدد أشكال الليشمانيا الطفلية، أدرجنا أنماط تقييد nagt من الـ 17 سلالة *المتاحة في المطبوعات* والأنماط المحددة بواسطة *عملية الهضم السيليكي* في ثلاثة متتابعات من قاعدة بيانات GenBank.

النتائج: قدمت سلالات الليشمانيا الطفلية *المغربية* مستوى معين من التنوع الجيني وتم تحديد ستة مورثات مميزة *من* RFLP-nagt تم تحديد ثلاثة أنواع من الطرز الوراثية الستة فقط في المجموعة المغربية *من نوع الليشمانيا الطفلية*: المتغير M1 (15%)، والمتغير M2 (7.5٪) ، والمتغير M3 (2.5٪). تم وصف النمط الوراثي الأكثر شيوعًا (65٪) ، والمتغير 2 (2.5٪) ، والمتغير 4 (7.5٪) سابقاً في عدة بلدان متوطن لديها داء الليشمانيات. قام تحليل تطور السلالات بتقسيم مجموعتنا *الليشمانيا الطفلية* إلى مجموعتين متميزتين ، في حين تم تمييز المتغير M2 بوضوح من كل من المجموعة الأولى والمجموعة الثانية. يسلط هذا التوزيع الضوء على درجة التباين الوراثي بين تجمعات *الليشمانيا الطفلية* المغربية.

الاستنتاج: أظهرت طريقة *تفاعل البوليميراز المتسلسل - تقييد طول الجزء متعدد الأشكال RFLP-PCR* nagt التي تم عرضها هنا تباينًا جينيًا مهمًا بين سلالات الليشمانيا الطفلية *المغربية* المعزولة من مستودعات البشر والكلاب في 6 تراكيب وراثية محددة. ثلاثة من التراكيب الوراثية *المغربية* الستة ، لم يسبق وصفها وتدعم التنوع الوراثي المعين لتجمعات *الليشمانيا الطفلية* المغربية المذكورة في دراسات أخرى..

Translated from English version into Arabic by Free bird, proofread by Saher Salama, through

TRANSLATORS
WITHOUT BORDERS

基于 *nagt* 基因 PCR-RFLP 技术分析人源和动物源婴儿利什曼原虫分离株的遗传多态性

Adil El Hamouchi, Sofia El Kacem, Rajaa Ejghal and Meryem Lemrani

摘要

引言：婴儿利什曼原虫（*Leishmania infantum*）是地中海地区人类内脏利什曼病（VL）和散发皮肤利什曼病（CL）的致病原。利什曼原虫的遗传变异可产生多种表型，这些表型与地理分布和临床表现多样性有关。本研究主要探讨摩洛哥不同地区人源和动物源婴儿利什曼原虫分离株的遗传多态性。

方法：针对编码 N-乙酰葡糖胺-1-磷酸转移酶的单拷贝（*nagt*）基因，采用 PCR-RFLP 方法分析 33 株人源（VL 31 株、CL 2 株）和 7 株犬源，共计 40 株摩洛哥婴儿利什曼原虫 MON-1 分离株的遗传多样性。为了更全面地分析，本研究还涵盖了文献提及的 17 个虫株的限制性酶切模式和 3 个 GenBank 数据库的电脑模拟酶切模式。
结果：从摩洛哥婴儿利什曼原虫虫株中鉴定出 6 种不同的 *nagt*-RFLP 基因型，表现出一定的遗传多样性。其中 3 种仅见于感染婴儿利什曼原虫的摩洛哥人群，即突变型 M1（15％）、突变型 M2（7.5％）和突变型 M3（2.5％）。其余的普通型（65％）、突变型 2（2.5％）和突变型 4（7.5％）在几个利什曼病流行国家中已有报道。系统发育分析显示，婴儿利什曼原虫群体分为两个不同的集簇，而突变型 M2 明显区别于集簇 I 和集簇 II。这种分布突出了摩洛哥婴儿利什曼原虫的遗传差异性。
结论：本研究所涉及的摩洛哥人源和犬源婴儿利什曼虫株可分为 6 个基因型，具有重要的遗传多样性。其中 3 个为首次报道，同其他研究报道共同反映出摩洛哥地区婴儿利什曼虫种群的遗传多样性。

Translated from English version into Chinese by Peng Song, edited by Pin Yang

CHINESE CENTER FOR DISEASE CONTROL AND PREVENTION
NATIONAL INSTITUTE OF PARASITIC DISEASES

Polymorphisme génétique d' isolats de *Leishmania infantum* prélevés sur des humains et des animaux, déterminé par *PCR-RFLP* sur le gène Nagt

Adil El Hamouchi, Sofia El Kacem, Rajaa Ejghal et Meryem Lemrani

Résumé

Contexte: *Leishmania infantum* est l'agent causal de la leishmaniose viscérale (LV) et, sporadiquement, de la leishmaniose cutanée (VC) humaine dans le bassin méditerranéen. La variabilité génétique des parasites*Leishmania* peut produire différents phénotypes liés à la distribution géographique et à la diversité des manifestations cliniques. Le principal objectif de cette étude était d'explorer le polymorphisme génétique *d'isolats de L. infantum* prélevés sur des hôtes humains et animaux dans différentes régions du Maroc.
Méthodes: La variabilité génétique intraspécifique de 40 souches de*L. infantum* MON-1 isolées sur des patients marocains souffrant de LV (n = 31) et de LC (n = 2) et sur des chiens (n = 7) a été évaluée par PCR-RFLP sur le gène*Nagt*à copie unique, codant pour la N-acétylglucosamine-1-phosphate transférase. Afin d'analyser plus exhaustivement le polymorphisme de *L. infantum* , nous avons inclus les schémas de restriction de *Nagt* de 17 souches qui ont fait l'objet de publication et des schémas déterminés par digestion *in silico* de trois séquences de la base de données GenBank.
Résultats: Les souches marocaines *de L. infantum* présentaient un certain niveau de diversité génétique, et nous avons identifié par RFLP six génotypes *distincts*de Nagt. Trois de ces six génotypes ont été identifiés exclusivement dans la population marocaine de *L. infantum*: les variants M1 (15 %), M2 (7,5 %) et M3 (2,5 %). Le génotype le plus commun (65 %), le variant 2 (2,5 %) et le variant 4 (7,5 %) ont déjà été décrits dans plusieurs pays où la leishmaniose est endémique. L'analyse phylogénétique a scindé notre population de *L. infantum* en deux clusters distincts, le variant M2 se distinguant clairement du cluster I comme du cluster II. Cette distribution met en lumière la forte variabilité génétique qui prévaut au sein de la population marocaine *de L. infantum* .
Conclusion: Avec 6 génotypes identifiés, la méthode par PCR-RFLP sur*Nagt* présentée ici a mis en évidence une forte hétérogénéité génétique parmi les souches marocaines de *L. infantum* isolées sur des réservoirs humains et canins. Trois des six génotypes marocains de *Nagt* n'ont pas été décrits précédemment, ce qui tend à confirmer la diversité génétique

particulièrement importante au sein de la population marocaine atteinte par la *L. infantum*, déjà signalée dans d'autres études.

Translated from English version into French by Suzanne Assenat, proofread by Karine H, through

Генетический полиморфизм изолятов *Leishmania infantum* у людей и животных с помощью *ПЦР-ПДРФ* с использованием NAGT

Адил эль Хамучи, София эль Касем, Раджаа Эджгхал и Мерием Лемрани

Аннотация

Справочная информация: *Leishmania infantum* является этиологическим фактором висцерального лейшманиоза (VL) и спорадического кожного лейшманиоза (CL) у человека в Средиземноморском регионе. Генетическое разнообразие паразитов рода *лейшмания* может обусловливать различные фенотипы, которые могут быть связаны с распределением по географическим регионам и различием клинических проявлений. Основная цель исследования заключалась в изучении генетического полиморфизма изолятов *L. infantum* в организме хозяина — человека и животного в различных регионах Марокко.

Методы: Внутривидовая генетическая изменчивость 40 марокканских штаммов *L. infantum* MON-1, выделенных у пациентов с VL (*n* = 31) и CL (*n* = 2), а также у собак (*n* = 7), оценивалась с помощью метода ПЦР-ПДРФ с использованием *NAGT*— однокопийного гена, кодирующего N-ацетилглюкозамин-1-фосфат-трансферазу. Для более полного анализа полиморфизма *L. infantum* мы включили рестрикционные шаблоны *NAGT* от 17 штаммов, имеющиеся в литературе, и шаблоны, определенные при расщеплении *in-silico* по трём последовательностям из базы данных GenBank.

Результаты: На основании полученных штаммов марокканской *L. infantum* были выявлены определенный уровень генетического разнообразия и шесть различных генотипов *NAGT*-ПДРФ. Три из шести генотипов были выделены исключительно в марокканской популяции *L. infantum*: вариант M1 (15%), вариант M2 (7,5%) и вариант M3 (2,5%). Наиболее часто встречающийся генотип (65%), вариант 2 (2,5%) и вариант 4 (7,5%), были ранее описаны в нескольких странах с эндемическим лейшманиозом. Филогенетический анализ разделил нашу популяцию *L. infantum* на два различных кластера, где вариант M2 четко отличался как от кластера I, так и от кластера II. Это распределение подчеркивает степень генетической изменчивости в марокканской популяции *L. infantum*.

Заключение: Представленный здесь метод ПЦР-ПДРФ с использованием *NAGT* показал значительную генетическую неоднородность среди марокканских штаммов *L. infantum*, выделенных у резервуаров человека и собаки при 6 выявленных генотипах. Три из шести марокканских генотипов *NAGT* не были описаны ранее и подтверждают особое генетическое разнообразие марокканской популяции *L. infantum*, описанное в других исследованиях.

Translated from English version into Russian by Anfeta, proofread by Liudmila Tomanek, through

Polimorfismo genético en cepas aisladas de *Leishmania infantum* de humanos y animales determinado por *nagt* PCR-RFLP

Adil El Hamouchi, Sofia El Kacem, Rajaa Ejghal y Meryem Lemrani.

Resumen

Antecedentes: el *Leishmania infantum* es el agente causante de la leishmaniasis visceral y la leishmaniasis cutánea en humanos en la región mediterránea.. La variación genética de los parásitos *Leishmania* puede resultar en diferentes fenotipos que pueden ser asociados con la distribución geográfica y la diversidad de las manifestaciones clínicas. El objetivo principal de este estudio era explorar el polimorfismo genético en cepas aisladas de *L. Infantum* de huéspedes humanos y animales en diferentes regiones de Marruecos.

Métodos: Se evaluó la variabilidad genética intraespecífica de 40 cepas aisladas, en Marruecos, de*L. infantum* del tipo MON-1, tomadas de pacientes con leishmaniasis visceral (*n* = 31) y cutánea (*n* = 2) y de perros (*n* = 7). Dicha evaluación fue hecha por un análisis PCR-RFLP de *nagt*: un gen de copia sencilla que codifica la transferase N-acetilglucosamina-1-fosfato. Para un análisis más completo sobre el polimorfismo del *L. infantum* , incluimos los patrones de restricción presentes en el *nagt* de 17 cepas disponibles en la literatura ypatrones determinados por la digestión *in-silico* de tres secuencias del banco de datos del GenBank.

Resultados: las cepas de*L. infantum* de Marruecos presentaron un cierto nivel de diversidad genética y se identificaron seis genotipos distintivos de *nagt*-RFLP. Tres de los seis genotipos fueron identificados exclusivamente en la población de marroquí de *L. infantum* presente en: la variante M1 (15%), la M2 (7.5%) y la M3 (2.5%). Los casos más comunes de genotipo (65%), la variante tipo 2 (2.5%) y la variante tipo 4 (7.5%), fueron ya descritos previamente en varios países con leishmaniasis endémica. Un análisis filogenético separó nuestra población de *L. infantum* en dos grupos muy distintivos, mientras que la variante M2 se distinguió claramente tanto del grupo I como del grupo II. Esta distribución resalta el grado de variabilidad genética entre la población marroquí de*L. infantum*.

Conclusión: El método *nagt* PCR-RFLP presentado aquí mostró una importante heterogeneidad genética entre cepas aisladas de *L. infantum* tomadas de huéspedes humanos y caninos de Marruecos, resultando en la identificación de 6 genotipos. Tres de los seis genotipos *nagt* de Marruecos no habían sido descritos anteriormente, y apoyan la particular diversidad genética del *L. infantum* en Marruecos, que ya había sido reportada en otros estudios.

Translated from English version into Spanish by Emiliano S. Grill, proofread by Patricia Cassoni, through

TRANSLATORS
WITHOUT BORDERS
