# Supplementary material for: Genetic polymorphism in Leishmania infantum isolates from human and animals determined by nagt PCR-RFLP
Source: Infect Dis Poverty. 2018 Jun 14;7:54. doi: 10.1186/s40249-018-0439-y (PMC6001066; doi:10.1186/s40249-018-0439-y)
Supplement: Supplementary file 2 — Table S1. List of strains of L. infantum and L. donovani previously genotyped as nagt variant by Waki et al. [34] and used in this work. (DOCX 13 kb) [file 40249_2018_439_MOESM2_ESM.docx]

**Additional file 2: Table S1** List of strains of *L. infantum* and *L. donovani* previously genotyped as *nagt* variant by Waki et al (2007) and used in this work

| **Species** | **WHO Code** | **Country of Origin** | **Disease** |
| --- | --- | --- | --- |
| *L. infantum* | HOM/TR/00/OG-VL | Turkey | VL |
|  | HOM/TR/03/Adana 7 | Turkey | CL |
|  | HOM/GR/70?/GH5 | Greece | CL |
|  | CANL/IR/04/IR2B | Iran | VL |
|  | HOM/FR/80/189 | France | VL |
|  | HOM/ES/81/260 | Spain | VL |
|  | HOM/CN/80/801 | China | VL |
|  | HOM/TN/90/DREP13 | Tunisia | CL |
|  | HOM/BR/82/BA-2, C1 | Brazil | VL |
|  | HOM/CN/50?/Bman | China | VL |
|  | HOM/SD/03?/VL2 | Sudan | VL |
|  | HOM/KE/84/NLB323 | Kenya | VL |
|  | HOM/CN/93/KXG-LIU | China | CL |
|  | IWUI/CN/87/KXG65 | China | N/A |
|  | HOM/CN/89/Shandong | China | VL |
| *L. donovani* | HOM/IN/96/JD | India | VL |
|  | HOM/LK/03/H9 | Sri Lanka | CL |

Hosts: CANL, wolf (*C. lupus pallipos*); HOM, *Homo sapiens*; IWUI, sand fly (*P. major wui*)

Countries of origin: BR, Brazil; CN, China; ES, Spain; FR, France; GR, Greece; IN, India; IR, Iran; KE, Kenya; LK, Sri Lanka; SD, Sudan; TN, Tunisia; TR, Turkey.

Diseases: VL, Visceral leishmaniasis; CL, Cutaneous leishmaniasis

N/A : not applicable
