# Supplementary material for: Genetic polymorphism in Leishmania infantum isolates from human and animals determined by nagt PCR-RFLP
Source: Infect Dis Poverty. 2018 Jun 14;7:54. doi: 10.1186/s40249-018-0439-y (PMC6001066; doi:10.1186/s40249-018-0439-y)
Supplement: Supplementary file 3 — Table S2. Cut positions of restriction enzymes tested by in-silico digestion of nagt (1405 bp) of L. infantum JPCM5. (DOCX 12 kb) [file 40249_2018_439_MOESM3_ESM.docx]

**Additional file 3: Table S2** Cut positions of restriction enzymes tested by *in-silico* digestion of *nagt* (1405 bp) of *L. infantum* JPCM5.

| Name | Frequency | Cut positions |
| --- | --- | --- |
| NaeI | 2 | 797–895 |
| AlwI | 2 | 257–1310 |
| NciI | 2 | 222–388 |
